# Supplementary material for: Single Cell Determination of 7,8-dihydro-8-oxo-2′-deoxyguanosine by Fluorescence Techniques: Antibody vs. Avidin Labeling
Source: Molecules. 2023 May 25;28(11):4326. doi: 10.3390/molecules28114326 (PMC10254197; doi:10.3390/molecules28114326)
Supplement: Supplementary file 1 [file molecules-28-04326-s001.zip › molecules-2326729-supplementary.pdf]

Single cell determination of 7,8-dihydro-8-oxo-2′-deoxyguanosine by fluorescence techniques: antibody vs avidin labeling.

Giusy Maraventano et al.

Supplementary Figures

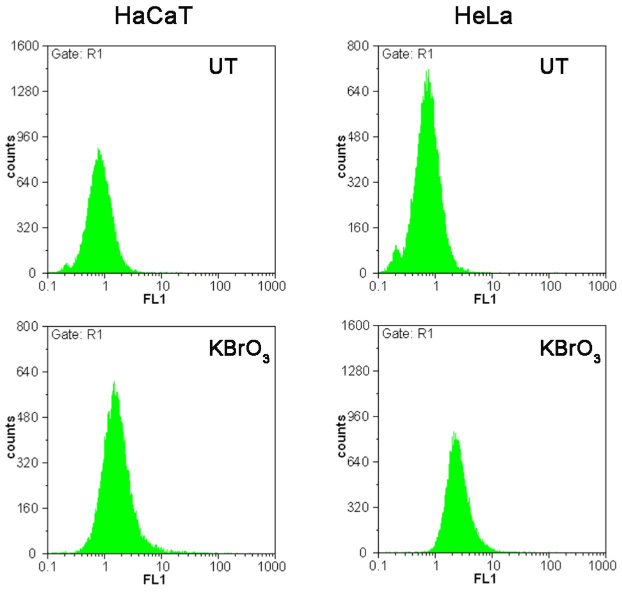

Supplementary Figure 1. Flow cytometric analysis of ROS production in HaCaT and HeLa cells lines, untreated (UT), or treated for 30 min with 40 mM $KBrO_3$. Detection was performed with the DCFH-DA assay. After treatment, cells were incubated for 30 min in 10 µM DCFH-DA in serum-free medium, then harvested and immediately measured with a Partec CyFlow flow cytometer.

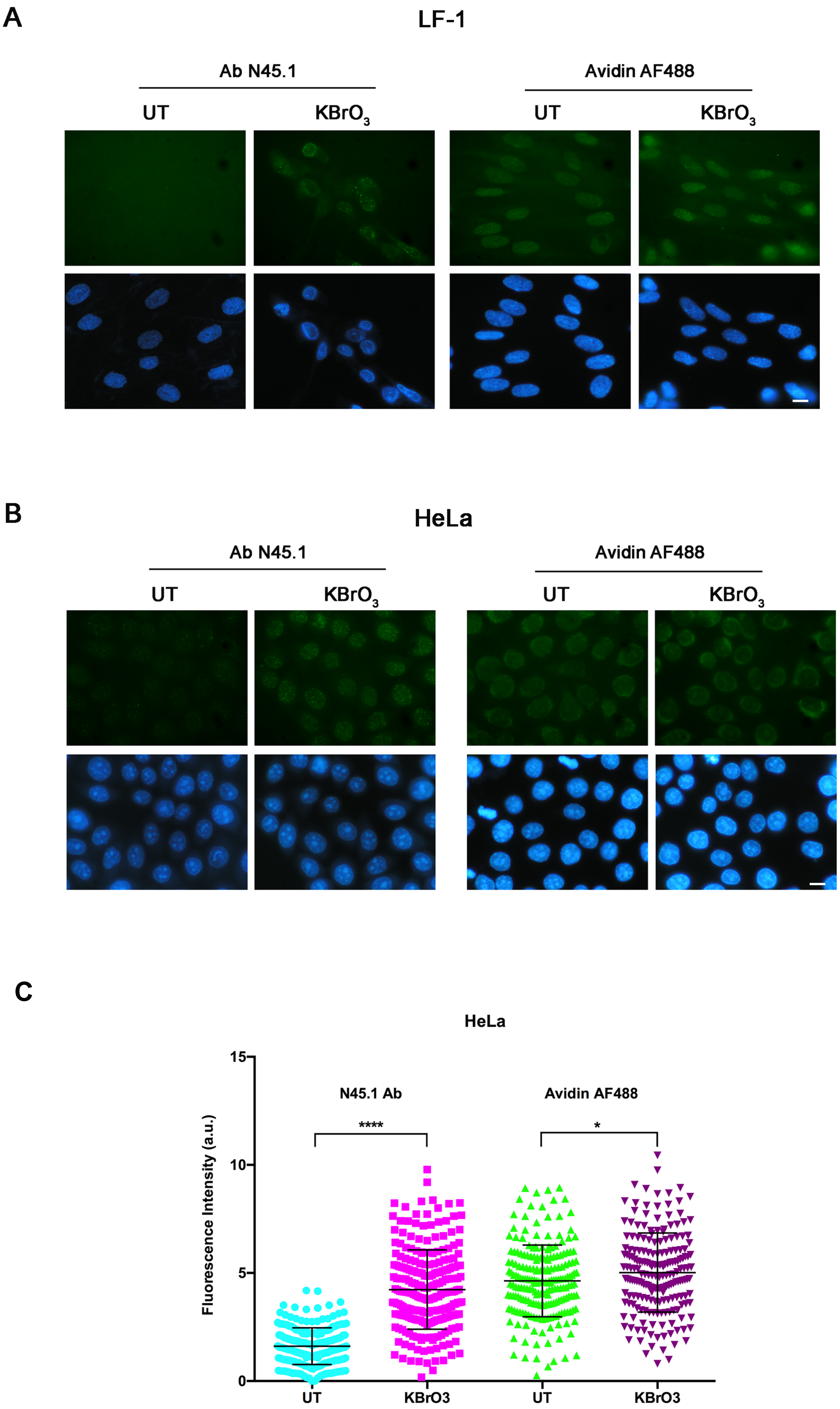
A
LF-1
Ab N45.1
Avidin AF488
UT
KBrO$_3$
UT
KBrO$_3$
B
HeLa
Ab N45.1
Avidin AF488
UT
KBrO$_3$
UT
KBrO$_3$
C
HeLa
Fluorescence Intensity (a.u.)
15
10
5
0
N45.1 Ab
Avidin AF488
****
*
UT
KBrO3
UT
KBrO3

Supplementary Figure 2. Fluorescence microscopy analysis of 8-oxodG in untreated cells (UT), or treated for 30 min with 40 mM $KBrO_3$. Detection was performed in LF-1 human fibroblasts (**A**), or in HeLa cells (**B**), after labeling with N45.1 antibody (Ab), or with avidin-AF488. Scale bar = 10 µm. (**C**) Quantification of fluorescence signals from HeLa cells stained with N45.1 antibody (Ab), or with avidin-AF488. At least 100 cells were measured for each sample. **** $P<0.001$; * $P<0.05$.

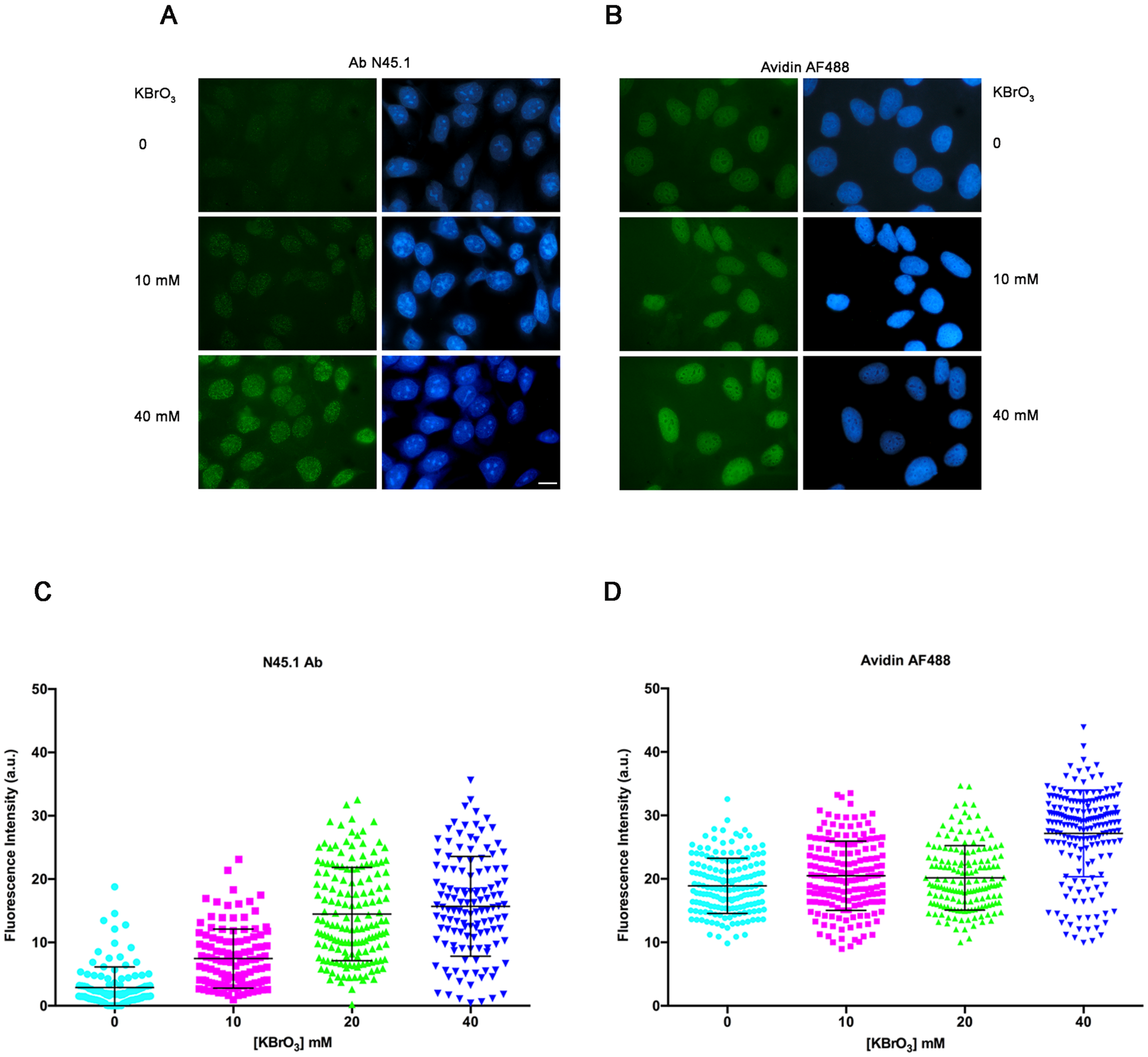

Supplementary Figure 3. Fluorescence images of 8-oxodG detected with Ab N54.1 (**A**) or with Avidin AF488 (**B**) in HaCaT cells untreated (0), or treated for 30 min with increasing concentrations of $KBrO_3$ (Scale bar = 10 µm). Quantification of fluorescence signals of 8-oxodG in HaCaT cells treated as above and labeled with N45.1 antibody (**C**), or with avidin-AF488 (**D**). At least 100 cells were measured for each sample.

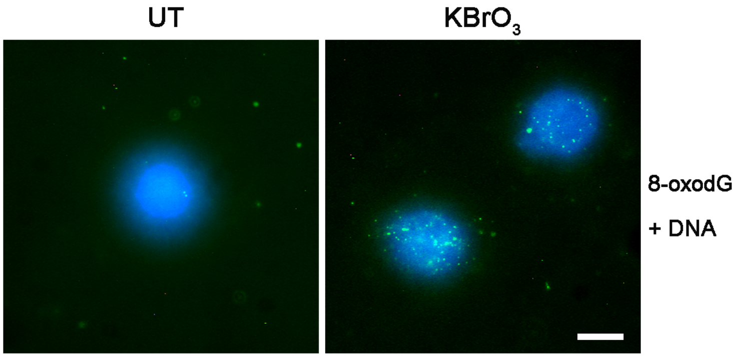

Supplementary Figure 4. Detection of 8-oxodG in lymphoblastoid cells untreated (UT), or treated for 30 min with 40 mM $KBrO_3$. Nucleoids were prepared as described in Materials and Methods, and labeling was performed with N45.1 antibody. Scale bar = 10 µm.
